# Precision Medicine Approaches to Diabetic Kidney Disease: Personalized Interventions on the Horizon

**DOI:** 10.7759/cureus.45575

**Published:** 2023-09-19

**Authors:** FNU Nageeta, Fahad Waqar, Ibtesam Allahi, Farhan Murtaza, Muhammad Nasir, FNU Danesh, Beena Irshad, Rajesh Kumar, Arslan Tayyab, Muhammad Saood Moazzam Khan, Satesh Kumar, Giustino Varrassi, Mahima Khatri, Muhammad Ali Muzammil, Tamam Mohamad

**Affiliations:** 1 Medicine, Ghulam Muhammad Mahar Medical College, Sukkur, PAK; 2 Medicine, Allama Iqbal Medical College, Lahore, PAK; 3 General Surgery, Allama Iqbal Medical College, Lahore, PAK; 4 Medicine, RHC Dhonkal, Gujranwala, PAK; 5 Internal Medicine, Liaquat University of Medical and Health Sciences, Thatta, PAK; 6 Medicine, Sharif Medical and Dental College, Lahore, PAK; 7 Spine Surgery, Sunnybrook Hospital, University of Toronto, Toronto, CAN; 8 Internal Medicine, Quaid-e-Azam Medical College, Bahawalpur, PAK; 9 Medicine and Surgery, Shaheed Mohtarma Benazir Bhutto Medical College, Karachi, PAK; 10 Pain Medicine, Paolo Procacci Foundation, Rome, ITA; 11 Medicine and Surgery, Dow University of Health Sciences, Karachi, PAK; 12 Internal Medicine, Dow University of Health Sciences, Karachi, PAK; 13 Cardiovascular Medicine, Wayne State University, Detroit, USA

**Keywords:** interventions, renal, dkd, kidney, diabetes

## Abstract

Diabetic kidney disease (DKD) is a significant complication of diabetes that requires innovative interventions to address its increasing impact. Precision medicine is a rapidly emerging paradigm that shows excellent promise in tailoring therapeutic strategies to the unique profiles of individual patients. This abstract examines the potential of precision medicine in managing DKD. It explores the genetic and molecular foundations, identifies biomarkers for risk assessment, provides insights into pharmacogenomics, and discusses targeted therapies. Integrating omics data and data analytics provides a comprehensive landscape for making informed decisions. The abstract highlights the difficulties encountered during the clinical implementation process, the ethical factors to be considered, and the importance of involving patients. In addition, it showcases case studies that demonstrate the effectiveness of precision-based interventions. As the field progresses, the abstract anticipates a future characterized by the integration of artificial intelligence in diagnostics and treatment. It highlights the significant impact that precision medicine can have in revolutionizing the provision of care for DKD.

## Introduction and background

Diabetic kidney disease (DKD) is a prominent microvascular complication of diabetes mellitus, playing a significant role in the worldwide prevalence of chronic kidney disease (CKD). With the global surge in diabetes cases, there is a growing prevalence of DKD, which calls for a thorough reassessment of its management strategies [1]. This section provides an overview of the complex landscape of DKD, including its prevalence and associated challenges. It then explores the concept of precision medicine and its potential to transform the management of DKD [1].

DKD: prevalence and challenges

Diabetes mellitus is a medical condition characterized by persistent high blood sugar levels, known as chronic hyperglycemia. It encompasses a range of metabolic disorders that can lead to damage in various organs of the body. Among these, DKD manifests as a standard and incapacitating complication, ultimately progressing to end-stage renal disease (ESRD) if not properly managed [2]. The epidemiological evidence highlights the increasing prevalence of DKD, with estimates indicating that approximately one-third of individuals with diabetes will develop DKD during their lifetime. In addition, DKD contributes to a considerable socioeconomic burden, leading to higher healthcare expenses and diminished quality of life. The adverse effects on patient well-being can be observed through hypertension, proteinuria, decreased glomerular filtration rate (GFR), and increased cardiovascular morbidity and mortality [3]. Despite the widespread recognition of the enormity of DKD, effectively managing this intricate condition remains a significant challenge. The diverse disease progression patterns and the variability in patient reactions to traditional treatments highlight the need for more personalized therapeutic approaches. Traditional treatments primarily target glycemic and blood pressure management. However, their effectiveness in preventing or slowing down the progression of DKD varies. This calls for a need to shift toward personalized interventions [4].

Precision medicine: an exemplary approach for customized interventions

Alternatively referred to as personalized medicine, precision medicine represents a significant paradigm shift in healthcare, moving away from a universal approach and embracing a customized, patient-focused strategy. This approach is based on recognizing that individuals demonstrate diverse susceptibility to diseases, and genetic, molecular, and environmental factors influence their responses to treatments [5]. Precision medicine aims to enhance treatment outcomes, mitigate adverse effects, and enhance patient compliance and quality of life by utilizing these factors. In the context of DKD, precision medicine represents a promising prospect. The current trial-and-error approach to treatment prescription may be replaced by targeted therapies tailored to the individual's specific genetic and molecular characteristics. This aspect holds significant relevance in DKD, considering the complex interaction between genetic predisposition and environmental triggers [6]. Through a comprehensive analysis of the genomic architecture of DKD, which encompasses susceptibility genes and epigenetic modifications, the field of precision medicine aims to identify individuals who are more susceptible to the disease. This knowledge enables the implementation of timely interventions that effectively mitigate the progression of the disease [7].

The relevance of precision medicine extends beyond genetic factors. Technological advancements, such as proteomics, metabolomics, and transcriptomics, have enabled the thorough profiling of patients. This allows for identifying subtle molecular signatures that indicate the onset or progression of diseases. Similar to diagnostic fingerprints, these signatures provide unparalleled insights into disease mechanisms and facilitate timely therapeutic interventions [8]. The fundamental principle of precision medicine is rooted in its capacity to provide tailored treatment protocols. Pharmacogenomics, the scientific study of genetic variations that impact drug responses, is crucial in this endeavor. For example, some individuals may have genetic polymorphisms that determine their response to renin-angiotensin-aldosterone system (RAAS) inhibitors, which are essential in managing DKD [9]. The practice of tailoring drug selections based on genetic markers holds the potential to optimize drug efficacy and minimize the occurrence of adverse reactions. The increasing prevalence of DKD and the emergence of the precision medicine paradigm indicate a promising future for diabetes care. The following sections of this review will provide a more comprehensive analysis of the genetic and molecular foundations of DKD and the rapidly developing area of biomarkers, pharmacogenomics, and emerging targeted therapies [10]. These various aspects collectively contribute to the emergence of a new era in which precision medicine provides personalized interventions that have the potential to reshape the course of DKD management, reduce its impact, and enhance patient outcomes.

## Review

Genetic and molecular basis of DKD: an investigation into susceptibility, pathways, and therapeutic implications

DKD is a commonly observed complication of diabetes mellitus that arises from a multifaceted interaction between genetic susceptibility and intricate molecular mechanisms. Gaining a comprehensive understanding of the genetic and molecular underpinnings of DKD is paramount in discovering new therapeutic approaches [1]. This knowledge will ultimately facilitate the development of precision medicine interventions. This section explores the genetic factors that contribute to the susceptibility of DKD. It also analyzes the molecular pathways that play a role in the development and progression of DKD. Furthermore, it highlights the potential of targeted therapies derived from these genetic and molecular findings [2]. 

Genetic Factors Influencing DKD Susceptibility

The heritability of DKD is apparent based on familial aggregation studies, indicating a significant genetic factor contributing to the susceptibility of this disease. Genome-wide association studies (GWAS) have played a crucial role in identifying genetic loci associated with the risk of DKD. These genetic loci frequently intersect with genes that have been implicated in the pathogenesis of diabetes, renal function, inflammation, and vascular homeostasis. An example of such a gene is the solute carrier family 12 member 3 (SLC12A3), which encodes the thiazide-sensitive sodium-chloride cotransporter. The presence of variants in the SLC12A3 gene has been associated with the development of diabetes and DKD [3]. These variants can potentially affect the regulation of sodium handling and blood pressure. Additional susceptibility genes implicated in DKD include protein kinase C-beta (PRKCB1), transforming growth factor-beta 1 (TGFB1), and tissue inhibitor of metalloproteinases 1 (TIMP1) [4]. These findings highlight the intricate genetic nature of DKD. In addition, it is worth noting that variations in the RAAS genes, specifically angiotensin-converting enzyme (ACE) and angiotensin II type 1 receptor (AGTR1), have a substantial influence on the susceptibility to DKD. These genes regulate blood pressure, renal hemodynamics, and fibrotic processes, which contribute to the complex pathophysiology of DKD [5].

Investigating Molecular Pathways in the Development and Progression of DKD

The pathogenesis of DKD involves a complex interplay of molecular pathways, including inflammation, oxidative stress, fibrosis, and endothelial dysfunction. Elevated glucose levels initiate intracellular signaling pathways, such as protein kinase C (PKC), nuclear factor-kappa B (NF-κB), and advanced glycation end products (AGEs), resulting in the development of inflammation and oxidative stress. Inflammatory cytokines, such as interleukin-6 (IL-6) and tumor necrosis factor-alpha (TNF-α), are crucial in coordinating immune cell infiltration and intensifying renal injury [6]. Oxidative stress, which is a characteristic feature of DKD, occurs as a result of mitochondrial dysfunction, increased production of reactive oxygen species (ROS), and compromised antioxidant mechanisms. ROS induce DNA damage, lipid peroxidation, and protein modifications, collectively contributing to the promotion of inflammation and endothelial dysfunction. Simultaneously, the dysregulation of crucial pathways, such as the mammalian target of rapamycin (mTOR) and the Janus kinase-signal transducer and activator of transcription (JAK-STAT) pathways, plays a role in glomerular hypertrophy, podocyte loss, and fibrosis. The activation of the TGF-β pathway is a critical factor in the progression of DKD [7]. This pathway serves as a master regulator of fibrosis. TGF-β induces the deposition of extracellular matrix and activation of myofibroblasts, ultimately leading to renal fibrosis and the impairment of functional nephrons. In addition, the dysregulation of the Wnt/β-catenin signaling pathway, which plays a crucial role in cell proliferation and differentiation, exacerbates tubulointerstitial fibrosis [8].

Exploring the Prospects of Targeted Therapies Utilizing Genetic and Molecular Findings

Exploring genetic and molecular factors underlying DKD deepens our comprehension of the mechanisms behind the disease. It presents opportunities for targeted interventions in precision medicine. Targeted therapies leverage these insights to improve specific disease components, ensuring optimal effectiveness and minimal adverse effects. Therapies targeting the RAAS demonstrate the promising capabilities of precision medicine [8]. The presence of genetic polymorphisms in ACE and AGTR1 genes serves to underscore the individual variations observed in the responsiveness of the RAAS. Therefore, ACE inhibitors and angiotensin II receptor blockers (ARBs) offer unique therapeutic opportunities for patients with specific genetic profiles, enhancing blood pressure management and reducing renal fibrosis. There is ongoing research into the potential use of anti-inflammatory agents, such as pentoxifylline and bardoxolone methyl, to mitigate the effects of inflammation in DKD. The potential of mTOR pathway inhibitors, such as sirolimus and everolimus, in reducing glomerular hypertrophy and proteinuria is evident. In a similar vein, antifibrotic agents, such as pirfenidone and nintedanib, are designed to specifically address fibrosis mediated by TGF-β, with the potential to mitigate structural damage to the kidneys [9].

The emergence of precision medicine aligns with the notion of personalized therapeutic regimens. Genetic profiling can identify patients who stand to benefit most from specific treatments, mitigating the trial-and-error approach that characterizes conventional therapies. In addition, identifying molecular signatures that indicate the onset and progression of diseases could inform early interventions, thereby helping prevent irreversible kidney damage [10]. The genetic and molecular foundations of DKD reveal a complex network of factors that contribute to susceptibility and regulate disease progression. The complex landscape depicted highlights the potential of precision medicine, which focuses on developing personalized therapies for patients. These tailored treatments aim to optimize therapeutic outcomes while reducing adverse effects. With the advancement of genetic insights and the unraveling of molecular mechanisms, there is a promising potential to revolutionize patient care and halt the progression of DKD [11]. This complication of diabetes poses a significant challenge, but with these developments, the horizon for DKD management appears brighter [11].

Exploring insights into omics technologies: biomarkers for early detection and risk stratification in DKD

Biomarkers are essential in understanding the complex nature of DKD, as they facilitate the early identification, risk assessment, and tailored patient interventions. This section provides an in-depth analysis of the importance of biomarkers in predicting the onset and progression of DKD, including both well-established and emerging candidates [12]. In addition, this study delves into the potential transformative capabilities of omics technologies, specifically genomics, proteomics, and metabolomics, in identifying innovative biomarkers. Incorporating biomarker-driven early detection into precision medicine highlights the necessity for customized interventions to mitigate the impact of DKD.

Onset and Progression of DKD: Established and Emerging Factors

Biomarkers are essential tools used to monitor the progression of diseases, predict outcomes, and inform therapeutic choices. Within the field of DKD, several well-established biomarkers have attracted significant attention due to their consistent correlation with the development and progression of the disease. Urinary albumin excretion, commonly referred to as albuminuria or proteinuria, is a fundamental biomarker used to assess the onset and progression of DKD [13]. Increased levels of urinary albumin not only indicate damage to the glomeruli but also serve as a predictor of cardiovascular risk. Another important indicator is the estimated glomerular filtration rate (eGFR), which assesses the decline in renal function. Nevertheless, it is essential to acknowledge the limitations of albuminuria and eGFR, such as their varying sensitivities and specificities. These limitations emphasize the need for new biomarkers that provide more accurate and detailed information. Promising advancements in the field of biomarkers include the emergence of neutrophil gelatinase-associated lipocalin (NGAL), kidney injury molecule-1 (KIM-1), and soluble urokinase-type plasminogen activator receptor (suPAR). These biomarkers show potential in enhancing the effectiveness of traditional markers. NGAL and KIM-1, secreted during tubular injury, offer valuable insights into the early stages of renal damage [14]. The suPAR, a marker of inflammation and immune activation, plays a significant role in risk stratification in DKD.

Omics Technologies: Deciphering the Complexity of Biomarkers

The advent of omics technologies, including genomics, proteomics, and metabolomics, has significantly transformed the field of biomarker discovery. These advanced technologies have provided researchers with the means to thoroughly investigate the molecular foundations of DKD. The field of genomics explores the genetic variations that may make individuals more susceptible to DKD [14-16]. On the other hand, proteomics focuses on understanding changes in protein expression and modifications. Lastly, metabolomics aims to identify alterations in metabolite profiles that can provide insights into the progression of the disease. Genomic studies have successfully identified genetic variants associated with susceptibility to DKD [12]. This breakthrough has allowed for the implementation of risk stratification strategies and the development of tailored interventions for at-risk individuals. Polymorphisms in genes, such as ACE, AGTR1, and SLC12A3, highlight individual variations in susceptibility to DKD. By comprehending these genetic factors, healthcare professionals can effectively identify individuals with an increased risk and proactively implement preventive measures [13].

Advanced mass spectrometry techniques in proteomics allow for identifying differentially expressed proteins in urine and serum. Studies in urinary proteomics have successfully identified potential biomarkers that indicate glomerular injury, including podocyte-derived proteins, such as nephrin and podocin. Furthermore, proteomics provides insights into the importance of inflammation, oxidative stress, and endothelial dysfunction in the pathogenesis of DKD, thereby identifying potential targets for therapeutic interventions. The metabolomics process, which involves analyzing metabolite concentrations, reveals disturbances in metabolic pathways crucial to the progression of DKD [6]. Altered lipid metabolism, amino acid imbalances, and dysregulated energy pathways emerge as critical players in DKD pathogenesis. These findings not only discover new biomarkers but also offer valuable insights into the underlying mechanisms of disease processes.

The Importance of Early Detection and Tailored Interventions

The incorporation of biomarkers into clinical practice facilitates the prompt identification of early DKD, enabling timely interventions to impede or alleviate the advancement of the disease. Early detection is pivotal, as interventions during the early stages of DKD hold the potential to preserve renal function and avert irreversible damage. Utilizing biomarker-driven risk stratification is instrumental in identifying high-risk individuals who stand to gain the most from intensified glucose and blood pressure control [17]. In addition, integrating biomarkers with precision medicine establishes the basis for tailored interventions. Integrating genetic profiles and biomarker insights allows for the customization of therapeutic regimens based on an individual's unique molecular composition. For example, genetic variations that impact drug responses can determine the choice of medications, thereby optimizing effectiveness and minimizing potential side effects [18].

Biomarkers play a crucial role in the fight against DKD by facilitating early detection, risk assessment, and tailored interventions. Integrating well-established and emerging biomarkers with advanced omics technologies, such as genomics, proteomics, and metabolomics, enables a comprehensive comprehension of disease mechanisms [19]. This comprehension, in turn, enables healthcare professionals to interpret the complex molecular aspects of DKD and apply focused interventions that have the potential to significantly transform the course of this challenging complication associated with diabetes. The early identification of DKD, guided by biomarker analysis, aligns with the principles of precision medicine. This approach promotes personalized interventions that effectively reduce the effects of DKD and improve patient outcomes [20].

Role of pharmacogenomics in understanding drug responses in DKD: customizing treatments based on genetic insights

Pharmacogenomics, a field of study investigating the impact of genetic variations on an individual's response to medications, represents a groundbreaking paradigm in treating DKD. This section explains the concept of pharmacogenomics and its implications for drug responses in patients with DKD. By examining drugs frequently utilized in treating DKD, we can ascertain the role of genetic variations in influencing diverse therapeutic outcomes [21]. In conclusion, the ability to customize drug regimens based on individual genetic profiles presents a promising opportunity to enhance the effectiveness of DKD treatment and minimize adverse outcomes.

Pharmacogenomics: The Influence of Genetic Variations on Drug Responses

Pharmacogenomics is based on the premise that genetic variations contribute to a wide range of drug responses, including differences in effectiveness and safety. These genetic variations can potentially impact drug metabolism, transport, receptor interactions, and subsequent cellular pathways [22]. Pharmacogenomics allows for the customization of drug regimens by analyzing these variations to optimize therapeutic outcomes and minimize adverse reactions. In the context of DKD, pharmacogenomics provides a comprehensive understanding of how genetic factors influence and modify drug responses. Considering the intricate nature of DKD and its multifactorial causes, it is crucial to incorporate genetic knowledge to improve treatment strategies' accuracy and effectiveness [13].

Genetic Influences on Drug Responses: Illustrative Cases and Implications

Numerous pharmaceutical agents have proven effective in the management of different aspects of DKD, such as the regulation of blood pressure, glucose levels, and reduction of proteinuria. However, the diversity in treatment responses can be ascribed to genetic variations that regulate drug metabolism and targets. One noteworthy illustration involves the utilization of RAAS inhibitors. ACE inhibitors and ARBs are widely recognized as essential treatments for managing DKD [18]. These medications are specifically designed to reduce proteinuria and decelerate the progression of the disease. Genetic polymorphisms can influence the efficacy of these drugs in the ACE and AGTR1 genes. The ACE insertion/deletion (I/D) polymorphism has been found to impact the response to ACE inhibitors, with individuals carrying the DD genotype exhibiting diminished effectiveness of treatment. Similarly, the presence of AGTR1 polymorphisms has been found to play a role in the variability of ARB responses [19].

In addition, it is worth noting that sodium-glucose cotransporter 2 (SGLT2) inhibitors, which are innovative antidiabetic medications known for their renoprotective properties, have been found to elicit diverse responses that genetic factors can influence. The SLC5A2 gene is responsible for encoding SGLT2, and genetic variations in this gene have been found to affect the response to certain drugs [20]. Variants, such as SLC5A2 rs311057, have been found to enhance the efficacy of SGLT2 inhibitors, highlighting the importance of pharmacogenomics in customizing treatment approaches.

Customizing Drug Regimens Based on Individual Genetic Profiles

The potential of pharmacogenomics resides in its ability to provide valuable insights for treatment decisions, enhancing therapeutic outcomes while mitigating adverse effects. Within the domain of DKD, this concept involves customizing drug treatment plans according to the specific genetic profiles of individuals. Genetic testing to evaluate pertinent polymorphisms enables healthcare professionals to predict how patients will respond to specific medications [21]. Patients with genotypes linked to reduced drug effectiveness or heightened susceptibility to adverse reactions can be directed toward alternative treatment strategies. For example, individuals who possess the DD genotype for ACE inhibitors may be recommended higher dosages or alternative medications. By contrast, those with favorable genotypes for SGLT2 inhibitors may be given priority for these treatment options [22]. 

In addition, pharmacogenomic insights can be applied to effectively manage the comorbidities frequently observed in patients with DKD. Given the interconnection between cardiovascular complications and DKD, it is essential to consider the impact of genetic factors on the effectiveness and safety of medications used to manage cardiovascular risk factors. This includes lipid-lowering agents and antihypertensives [23]. Pharmacogenomics represents a patient-centered approach to treating DKD, in which genetic variations are utilized as guiding tools for therapeutic decision-making. The complex interplay among genetic composition, drug metabolism, and target interactions significantly influences treatment outcomes. Pharmacogenomic insights play a crucial role in connecting the variability in drug effectiveness with personalized interventions. By identifying genetic predictors of drug responses, clinicians can avoid the trial-and-error approach to treatment for DKD [24]. Customized drug regimens that are optimized based on individual genetic profiles provide a promising approach to enhance the effectiveness of treatments, minimize the occurrence of adverse reactions, and promote better patient adherence. In the field of DKD, which is known for its complex causes and diverse nature, pharmacogenomics plays a crucial role in developing precision medicine strategies. These strategies aim to improve treatment outcomes and enhance the quality of care for individuals facing this challenging complication of diabetes [25].

Advancements in therapeutic approaches and precision medicine for DKD: harnessing innovation for enhanced patient care

The management of DKD is undergoing significant changes as it embraces innovative strategies to address its complex challenges [25]. This section explores various treatment strategies, including current standards and advanced interventions, such as targeted monoclonal antibodies (mAbs), gene therapies, and RNA-based interventions. Moreover, this study explores the realm of clinical trials, uncovering the encouraging results that emerge from implementing precision medicine-based strategies in managing DKD.

Present Standard Treatments for DKD

The treatment of DKD typically focuses on achieving glycemic control and managing blood pressure to minimize the advancement of renal damage. ACE inhibitors and ARBs are widely recognized as fundamental components of therapy. These medications exert renoprotective effects by modulating the RAAS [26]. The primary objective of these agents is to target the reduction of proteinuria and control blood pressure, which collectively contribute to slowing down the deterioration of renal function. In addition, it is crucial to incorporate blood pressure management into caring for patients with DKD. This involves diuretics, calcium channel blockers, and beta-blockers, as hypertension can worsen renal damage. To optimize the management of DKD, it is important to incorporate lifestyle modifications, such as dietary sodium restriction, regular exercise, and weight management [13-16]. These modifications work together with pharmacological interventions to achieve the best patient outcomes.

Investigating Innovative Therapeutic Strategies

The development of new therapeutic paradigms presents innovative approaches for managing DKD to implement precise and targeted interventions to modify the progression of the disease.

Targeted mAbs: mAbs have been developed to selectively target specific molecules that play a role in the pathogenesis of DKD. Agents, such as anti-vascular endothelial growth factor (VEGF) mAbs, have been shown to mitigate the vascular abnormalities associated with DKD, thereby preserving renal function [25-28]. In addition, mAbs that specifically target TGF-β have effectively reduced fibrotic processes, thereby relieving glomerulosclerosis and tubulointerstitial fibrosis. Gene therapies involve the administration of therapeutic genes to regulate pathways associated with diseases. Within the field of DKD, gene therapies show potential in the regulation of renoprotective mechanisms. For example, administering genes that encode growth factors, such as hepatocyte growth factor (HGF) and VEGF, facilitates renal regeneration and angiogenesis, thereby mitigating the fibrotic cascade. Furthermore, it has been observed that gene therapies aimed at the RAAS have the potential to effectively mitigate the pathological activation of RAAS observed in DKD. RNA-based interventions, including small interfering RNAs (siRNAs) and antisense oligonucleotides (ASOs), can induce gene silencing or upregulation, respectively. In the context of DKD, the use of siRNAs that specifically target molecules promoting fibrosis, such as connective tissue growth factor (CTGF), has been observed to impede the progression of fibrosis. ASOs that modulate RNA splicing show promise in regulating crucial molecules implicated in the pathogenesis of DKD [28].

Clinical trials and precision medicine-based interventions are subjects of interest and importance in healthcare. These trials and interventions involve rigorous scientific research and analysis to evaluate the effectiveness and safety of medical treatments and interventions. Precision medicine, in particular, focuses on tailoring medical interventions to individual patients based on clinical trials, which are essential platforms for evaluating the safety and effectiveness of emerging therapeutic strategies, serving as catalysts for innovations in the field [29]. The trials prominently showcase precision medicine, which is supported by genetic insights. This signifies the advent of a new era in targeted treatments for DKD.

Bardoxolone methyl: Bardoxolone methyl, a recently developed antioxidant inflammation modulator (AIM), has attracted significant interest due to its potential to reduce inflammation and oxidative stress in DKD. Various clinical trials, such as the BEACON study, have provided evidence of positive outcomes in terms of eGFR enhancement and reduction of albuminuria [30]. The significance of precision medicine in optimizing patient selection is highlighted by the genetic markers that influence the response to bardoxolone methyl.

SGLT2 inhibitors: SGLT2 inhibitors, initially developed to manage diabetes, have demonstrated renoprotective properties. The medications empagliflozin and canagliflozin have been shown to reduce cardiovascular events and improve renal outcomes in patients with DKD based on the EMPA-REG OUTCOME and CREDENCE trials, respectively. Genetic markers, such as the polymorphism SLC5A2 rs311057, have been found to impact the responses to SGLT2 inhibitors. This discovery helps to uncover the genetic basis underlying the effectiveness of these treatments [30].

Anti-fibrotic therapies: Emerging anti-fibrotic agents, such as pirfenidone and nintedanib, are revolutionizing the treatment field. Pirfenidone, a pharmacologically approved anti-fibrotic agent used to treat idiopathic pulmonary fibrosis, has demonstrated potential in mitigating renal fibrosis by inhibiting the TGF-β pathway. Nintedanib, known for its anti-fibrotic properties in multiple organs, shows potential to slow down the progression of DKD [12]. Clinical trials examining these agents, such as the PIRFENIDONE study, shed light on the future of interventions for DKD guided by precision medicine.

The convergence of emerging therapies and precision medicine-driven approaches is shaping the future of treatments for DKD. The shift from traditional standards to targeted mAbs, gene therapies, and RNA-based interventions signifies a significant change toward personalized and mechanistically nuanced treatments. The effectiveness of these new interventions is supported by clinical trials, highlighting the importance of incorporating genetic knowledge to enhance therapeutic results [14-16]. The utilization of genetic markers in patient selection and treatment customization has the potential to bring about a new era of precision medicine in the management of DKD. This holds great promise in altering the course of this intricate condition. These emerging therapies collectively represent a source of optimism, shedding light on a potential path toward improved patient care and better outcomes in the complex landscape of DKD [16].

Challenges and ethical considerations associated with the implementation of precision medicine for DKD: addressing the intricate landscape

Precision medicine represents a transformative phase in healthcare, offering customized interventions to achieve the most favorable results for patients. However, the implementation of this approach within the context of DKD poses a variety of complex challenges and ethical considerations. This section explores the complex challenges that hinder the implementation of precision medicine for DKD. These challenges encompass issues, such as data privacy, cost, and accessibility, as well as the ethical considerations surrounding genetic testing, data sharing, and informed consent. By acknowledging and addressing these challenges and ethical dilemmas, we are laying the foundation for a more thoughtful and fair incorporation of precision medicine into managing DKD [30].

Challenges in the Implementation of Precision Medicine for DKD

Data privacy and security are crucial considerations in precision medicine, as it heavily relies on the meticulous gathering, safekeeping, and examination of patients' genetic and medical information. Preserving privacy and security for this susceptible information presents a considerable challenge. It is imperative to protect patient confidentiality by implementing solid data encryption, implementing controlled access measures, and adhering to privacy regulations, such as the Health Insurance Portability and Accountability Act (HIPAA) in the United States [12]. Achieving a harmonious equilibrium between facilitating data accessibility for research purposes and safeguarding individual privacy continues to be a nuanced undertaking. Cost and resource constraints are essential considerations in precision medicine, as the advanced technologies and analyses involved in this approach can be associated with higher expenses. Sequencing genomes, conducting omics analyses, and creating personalized treatment plans can impose significant financial challenges for patients and healthcare systems. Addressing the challenge of balancing cost-effectiveness with delivering high-quality care presents a complex dilemma [13]. The achievement of precision medicine's potential relies heavily on ensuring equal access for all patient populations, thereby addressing issues of accessibility and disparities. Nevertheless, disparities in healthcare infrastructure, access to technology, and socioeconomic status can further exacerbate pre-existing health inequalities. To ensure that precision medicine is beneficial to a wide range of demographics, it is imperative to address barriers to accessibility and incorporate culturally sensitive practices [15].

Ethical Considerations in Precision Medicine for DKD

The utilization of genetic testing plays a crucial role in precision medicine, as it allows for the identification of markers related to disease predisposition and therapeutic response. Genetic testing raises ethical concerns, specifically about the issue of informed consent. Patients must have a comprehensive understanding of the implications associated with genetic testing. This includes being aware of the potential insights it can provide into other health conditions and the possibility of uncovering non-paternity or non-maternity situations. Ensuring the provision of comprehensive, lucid, and culturally sensitive information during the consent process is of utmost importance [16]. 

Data sharing and privacy: The progress of precision medicine relies on exchanging genetic and medical data among various institutions, researchers, and consortia. The acceleration of discoveries through data sharing has brought to light ethical concerns regarding patient consent for using data beyond the original study's intended scope. Finding a harmonious equilibrium between facilitating advancements in research and upholding patients' autonomy regarding their data presents a multifaceted ethical dilemma [18]. The disclosure of genetic information can potentially result in unintended discrimination or stigmatization, particularly in cases where genetic predispositions to diseases, such as DKD, are identified. Individuals may be treated differently by employers, insurers, and social circles based on their genetic risk factors. Legislations, such as the Genetic Information Nondiscrimination Act (GINA) in the United States, provide safeguards against genetic discrimination. However, it is imperative to maintain diligent oversight of these ethical considerations [10].

Equity and inclusivity: Equity and inclusivity are crucial considerations in advancing precision medicine, as it is imperative to ensure equal access to its benefits ethically. It is imperative to ensure proper representation of diverse populations in research studies in order to prevent the perpetuation of health disparities. Insufficient representation can result in biased treatments and algorithms that disproportionately benefit specific demographics, amplifying pre-existing inequalities. Incorporating precision medicine into managing DKD involves various challenges and ethical considerations [25]. Resolving these complexities necessitates a collective endeavor involving healthcare professionals, policymakers, researchers, and patients. To address the challenges and uphold ethical principles, it is recommended to employ strategies, such as implementing robust data protection mechanisms, designing affordable care models, and advocating for comprehensive informed consent. As we confront these challenges and ethical dilemmas, it is imperative to maintain an unwavering dedication to equity and inclusivity. Precision medicine should be accessible to all individuals, irrespective of their background, to ensure that everyone can take advantage of the latest advancements in DKD management.

In summary, precision medicine can potentially transform the field of DKD management significantly [30]. However, the successful integration of this endeavor relies on our capacity to overcome challenges and navigate ethical complexities with prudence and empathy. By implementing this approach, we demonstrate our commitment to upholding the principles of patient autonomy, privacy, and justice as we strive to advance the field of care for DKD for the benefit of all individuals impacted by this debilitating complication of diabetes [22].

Clinical implementation and patient engagement in precision medicine for DKD: facilitating the journey toward individualized care

Implementing precision medicine in clinical practice significantly advances DKD management. This section explores the strategies utilized to incorporate precision medicine approaches into clinical care, focusing on patient education and engagement in the decision-making process [26]. Moreover, the analysis explores the elucidation offered by case studies and real-world illustrations, demonstrating practical implementations of precision medicine in managing DKD. The conversation delves into the domain of prospects, including artificial intelligence (AI)-powered diagnostics and therapies, ongoing research endeavors, and the crucial impact of precision medicine on reshaping the treatment of DKD and improving patient outcomes [27].

Strategies for the Implementation of Precision Medicine in Clinical Practice

The successful implementation of precision medicine, moving from theoretical concepts to practical clinical advantages, requires establishing a comprehensive framework. This framework facilitates the comprehensive integration of genomics, omics data, and clinical parameters to customize treatment regimens for individual patients. Health informatics systems, such as electronic health records (EHRs), play a crucial role in facilitating the smooth data exchange [18]. This capability empowers clinicians to make well-informed decisions by leveraging comprehensive patient profiles. In addition, the formation of multidisciplinary teams consisting of clinicians, geneticists, data scientists, and ethicists promotes collaboration and the sharing of expertise, thereby facilitating the effective integration of precision medicine. By integrating clinical insights with cutting-edge technological capabilities, this approach is poised to transform care provision for patients with DKD [30].

Patient Education and Engagement: Facilitating Empowered Decision-Making Through Information

It is crucial to provide patients with comprehensive information regarding precision medicine's principles, benefits, and limitations to empower them and facilitate informed decision-making. Patient education facilitates the development of comprehension regarding genetic testing, personalized treatment alternatives, and potential results. Patients who possess this knowledge are more prepared to actively participate in shared decision-making actively, thereby enhancing the optimization of treatment plans that align with their values, preferences, and goals [25]. The practice of shared decision-making improves treatment adherence. It can significantly increase patient satisfaction and cultivate a greater sense of patient agency. Healthcare professionals who assume the role of educators and facilitators are crucial in actively empowering patients to participate in their healthcare journey. This collaborative partnership between clinicians and patients fosters a mutually beneficial relationship, ultimately improving treatment outcomes and enhancing the overall patient experience.

Utilizing Case Studies and Real-World Examples to Harness the Potential of Precision Medicine

Precision medicine has emerged as a promising field with the potential to revolutionize healthcare [16]. To fully understand and leverage this potential, it is crucial to examine case studies and real-world examples demonstrating precision medicine's practical applications and impact. By analyzing case studies, we can gain valuable insights into the successful implementation of

Precision medicine's utilization in managing DKD as demonstrated through compelling case studies and real-world illustrations

Case Study 1: Selection of SGLT2 Inhibitors

In this case study, we will discuss selecting SGLT2 inhibitors. A patient diagnosed with DKD arrives at the clinic. Genetic testing results indicate the presence of the SLC5A2 rs311057 polymorphism, which has been linked to an increased response to SGLT2 inhibitors. Based on this information, clinicians decide to prescribe an SGLT2 inhibitor, which significantly decreased blood glucose levels, proteinuria, and blood pressure [11]. The patient's response highlights the importance of genetic insights in informing therapeutic decisions, enhancing treatment outcomes, and mitigating the likelihood of adverse events.

Case Study 2: Personalization of Anti-Fibrotic Therapy

A patient diagnosed with DKD, who has not shown a positive response to traditional treatment methods, undergoes a thorough analysis of omics data. The results of the metabolomic analysis indicate an elevation in the concentrations of specific biomarkers associated with fibrosis. With the knowledge gained from these insights, clinicians commence anti-fibrotic therapy customized to match the patient's molecular signature [21]. Over time, the patient demonstrated notable enhancements in proteinuria, eGFR, and renal function, thereby highlighting the potential of precision medicine in identifying focused interventions for challenging cases.

Future directions and outlook

Cutting-edge advancements and continuous research endeavors are currently illuminating the field of precision medicine in DKD.

AI-Driven Diagnostics and Therapies

AI enhances the process of diagnostics by efficiently analyzing intricate omics data, providing insights into disease mechanisms, and identifying potential targets for therapeutic interventions [25]. AI-driven predictive algorithms provide personalized treatment predictions, assisting clinicians in selecting interventions with the highest probability of achieving positive outcomes.

Current Research and Unexplored Areas

Continual research efforts consistently reveal novel genetic markers, molecular pathways, and therapeutic targets, thereby enhancing the profound influence of precision medicine. Emerging fields, such as epigenomics, single-cell sequencing, and systems biology, present promising opportunities for managing DKD. Continued research into the genetic and molecular foundations of different subtypes of DKD, along with their corresponding responses to treatment, can potentially transform the field significantly [26].

In summary, the transition of precision medicine from a conceptual idea to a practical approach represents a significant milestone in the care for DKD. Implementing strategies focused on integration, patient education, and engagement contributes to a comprehensive approach to personalized care, ultimately enhancing the effectiveness of treatments and improving patient experiences. Case studies highlight the concrete advantages of precision medicine, influencing treatment modifications and resulting in favorable results [28]. The continuous advancements in AI-driven diagnostics and therapies, along with ongoing research efforts, are driving the progress of the precision revolution. The potential of precision medicine to revolutionize the treatment of DKD and improve patient outcomes is becoming increasingly evident as the field advances. Precision medicine represents a promising approach that sheds light on a trajectory toward enhanced, focused, and empathetic care for DKD, instilling optimism for the days ahead [30].

## Conclusions

The integration of precision medicine into clinical practice has the potential to significantly transform the management of DKD, effectively bridging the divide between conventional methods and personalized care. Patient education and engagement are recognized as crucial elements, promoting a collaborative partnership that empowers patients and enhances treatment outcomes. The case studies presented provide vivid depictions that effectively illustrate the tangible benefits of precision medicine. These examples substantiate precision medicine's efficacy in tailoring interventions and ultimately improving patient well-being. With the horizon in sight, the incorporation of AI-driven diagnostics and the advancement of research avenues enhances the potential of precision medicine to revolutionize care for DKD further. Within the expansive realm of healthcare, precision medicine represents a significant shift in approach. It embodies a patient-centric and scientifically sophisticated form of care that promises to transform the treatment of DKD and improve patient outcomes. This journey exemplifies the coming together of scientific progress, empathetic healthcare, and patient empowerment, with the potential to improve the quality of life for numerous individuals grappling with DKD.
